# Cost-effectiveness of physical activity-oriented interventions for improving mental health: a systematic review

**DOI:** 10.1186/s12889-025-22207-3

**Published:** 2025-05-13

**Authors:** Noara Alhusseini, Tracy Kuo Lin, Kalin Werner, George Lin, Yasmin Altwaijri, Baian A. Baattaiah, Tim Bruckner, Reem AlAhmed, Abdulrahman Alkabbani, Reem F. Alsukait, Volkan Cetinkaya, Hazzaa M. Al-Hazzaa, Saleh A. Alqahtani

**Affiliations:** 1 College of Medicine, Alfaisal University, Riyadh, Saudi Arabia; 2 Institute for Health & Aging, Department of Social and Behavioral Sciences, University of California, San Francisco, San Francisco, CA USA; 3 Department of Psychiatry and Behavioral Sciences, Stanford University School of Medicine, Stanford, CA USA; 4 Biostatistics, Epidemiology and Scientific Computing Department, King Faisal Specialist Hospital & Research Center, Riyadh, Saudi Arabia; 5 Department of Physical Therapy, Faculty of Medical Rehabilitation Sciences, King Abdulaziz University, Jeddah, Saudi Arabia; 6 Public Health, Center for Population, Inequality and Policy, University of California, Irvine, Irvine, CA USA; 7 Department of Medicine, King Faisal Specialist Hospital & Research Center, Riyadh, Saudi Arabia; 8 World Bank Group, Washington, D.C USA; 9 Lifestyle and Health Research Center, Health Sciences Research Center, Princess Nourah Bint, Riyadh, Saudi Arabia; 10 School of Sport Sciences, University of Jordan, Amman, Jordan; 11 Liver, Digestive, and Lifestyle Health Research Section, and Organ Transplant Center of Excellence, King Faisal Specialist Hospital & Research Center, Riyadh, Saudi Arabia; 12 Division of Gastroenterology & Hepatology, Weill Cornell Medicine, New York, NY USA

**Keywords:** Exercise, Physical activity, Mental health, Cost-effectiveness, Incremental cost-effectiveness ratio

## Abstract

**Question:**

Are physical activity-oriented interventions for improving mental health conditions cost-effective? This study systematically identified and summarized published evidence on the cost-effectiveness of physical activity-oriented interventions for improving mental health conditions.

**Study selection and analysis:**

We searched four databases (PubMed/Medline, Science Direct, PsychArticles, PsychINFO) for published studies (from any time and region) that (1) assessed physical activity-oriented interventions in mental health disorders, (2) undertook a full economic evaluation (and specifically cost-effectiveness analysis or cost-utility analysis), and (3) were in English. Data were extracted from included studies using a predetermined 32-item matrix using the Covidence software platform.

**Findings:**

Search and screening resulted in 11 studies eligible for inclusion. The incremental cost-effectiveness ratio ranged from £119 to £152,822 per quality-adjusted life year (QALY) gained. Physical activity interventions, including group sessions, such as dance exercise and walking, or one-on-one support through telephone or web-based personalized support and guidance, were found to be cost-effective. However, over half of the interventions (55%) were considered to be not cost-effective.

**Conclusions:**

Our review found that the current evidence is insufficient to conclude whether physical activity-oriented interventions for mental health are a cost-effective when compared with the standard of care of other treatment types. Better designed studies focusing on specific mental health conditions and physical activity interventions that address cost-effectiveness analysis are warranted. Physical activity-oriented interventions for improving mental health should adopt low-cost implementation strategies and include behavioral economics components.

**Supplementary Information:**

The online version contains supplementary material available at 10.1186/s12889-025-22207-3.

## Background

Mental disorders are considered one of the leading causes of disease burden [1]. Globally, between 1990 and 2019, the number of disability-adjusted life years (DALYs) due to mental disorders increased from 80.8 million to 125.3 million—while the proportion of global DALYs attributed to mental disorders increased from 3.1% to 4.9% [2]. The global costs of these disorders were estimated at USD 2.5 trillion in 2010 and are projected to more than double by 2030 [3]. 

Mental health costs are high; however, given the cost-effectiveness of interventions, improving mental health could lead to significant resource savings [1]. The growing burden of mental disorders calls for increased investment in related services to combat these conditions. It is crucial to efficiently allocate available healthcare resources. As such, cost-effectiveness analyses (CEA) and cost-utility analyses (CUA)—economic evaluations that are often leveraged by decision-makers to inform evidence-based decisions and allocate a budget efficiently to support cost-effective interventions [4, 5]—have been increasingly conducted in the field of mental health interventions [6]. 

Physical activity interventions—defined as any intervention including bodily movement produced by skeletal muscles that require energy expenditure—targeted for mental health could achieve comprehensive and positive outcomes. For instance, having structured exercise programs, including aerobic activities, strength training, and yoga, can positively influence overall well-being and mental health [7]. Such interventions can play a role in reducing stress hormones, releasing endorphins, improving the quality of sleep, and enhancing the sense of self-efficacy. Physical activity has been correlated with reducing stress, anxiety, and depression symptoms [8]. 

Concomitantly, it is important to identify cost-effective interventions that can be personalized according to a patient’s condition and economic status. Physical activities and exercise have been highlighted as a relatively low-cost intervention for various disease conditions [9]. Tailored exercise programs are found to reduce low back pain [10], reduce cardiac deaths [11], and improve health-related quality of life in patients with heart failure [12]. Physical activity and exercise are found to alleviate side effects in patients and survivors of breast cancer [13] and improve cardiorespiratory fitness during and after cancer treatment, symptoms, and physiologic effects during treatment [14]. 

Physical activity (PA) interventions for mental health can be more cost-effective in comparison to traditional mental health interventions such as (cognitive behavioral therapy [CBT] and pharmacological). CBT usually requires a particular clinical infrastructure, specialized personnel, and long-term appointments [15]. PA can be easily accessible, particularly in a low-source setting. It can also be self-managed or through community groups [16]. It offers benefits without the side effects associated with medication. It is important to note that while PA cannot replace the needed therapy for mental health, it may supplement therapy and in certain cases be suitable for individuals resisting therapy [17]. 

There is an abundance of studies and systematic reviews discussing the effects of physical activity on mental health, from mechanisms by which physical activity may improve mental health [18] to interventions that may prevent or improve various categories of mental disorders, including anxiety [19], depression [20], psychotic disorders [21], schizophrenia [22, 23], post-traumatic stress disorder [24], and self-esteem in children and adolescents [25–27]. However, to our knowledge, we know of no systematic reviews focusing on the cost-effectiveness of physical activity-oriented interventions on mental health. Systematically evaluating the evidence for cost-effective physical activity-oriented interventions for improving mental health conditions will help health sectors allocate and plan resources.

## Objective

The aim of this systematic review is to systematically identify and summarize published evidence on the cost-effectiveness of physical activity-oriented interventions for improving mental health conditions, including anxiety, depression, and psychotic disorders.

## Study selection and analysis

### Study design

We conducted a literature review following Preferred Reporting Items for Systematic Reviews and Meta-Analyses (PRISMA) reporting standards and registered with PROSPERO (CRD42023390930) [28]. We systematically searched four databases of published literature (PubMed/Medline, Science Direct, PsychArticles, PsychINFO) to identify published evidence on cost-effectiveness of physical activity-oriented interventions for ameliorating mental disorders. The following concepts were used in the development of a search string: (1) depression, (2) anxiety disorder, (3) post-traumatic stress disorder, (4) schizophrenia, (5) bipolar disorder, (6) cost-effectiveness, (7) physical activity, and (8) exercise. The search strategy is available in Supplementary Table S1.

### Ethics

We registered this study with PROSPERO (CRD42023390930). As a desk-based study with no human subject involvement, no ethics approval was required. Consent to Participate is also not applicable to this study.

### Eligibility criteria

Eligible studies met the following criteria for inclusion; they were (1) available in English, (2) assessed physical activity-oriented interventions in mental health disorders with physical activity referring to any bodily movement produced by skeletal muscles that require energy expenditure—including but not limited to exercise, walking, and sports. Mental health interventions were interventions for those with dysregulation in emotion and behavior (3) undertook a full economic evaluation comparing both costs and consequences (specifically CEA or CUA), including simulation models. All economic perspectives and effectiveness measures were included in our review. No limitations were placed on the age of study participants. Studies focusing on eHealth, exergames, and other remote interventions were not included in the review. In regards to mental disorders, we focused on those related to dysregulation in emotion and behavior (e.g., depression, schizophrenia, post-traumatic stress disorder) [29] and excluded neurocognitive conditions related to cognitive impairment (e.g., dementia). However, we adopted an inclusive approach with regard to the background and setting of studies, and we included all relevant studies that addressed a mental health outcome. For example, if a cost-effectiveness analysis study examined an intervention that addressed symptoms or comorbid mental disorders of individuals experiencing cognitive impairment, it was included in our review. In other words, mental disorders need not be the primary diagnosis, so long as the studied intervention addresses mental disorders, the study was included in the review.

Literature reviews and meta-analyses were not included in our final review; however, their references were searched for potentially appropriate studies. Studies that did not undergo peer review and gray literature materials were excluded. Similarly, studies without empirical data, conference abstracts, posters, or protocols were excluded from the review.

Duplicates were firstly removed using Covidence review software (Veritas Health Innovation, Melbourne, Australia. Available at www.covidence.org), and secondarily manually checked by the review team. For each study, two of the six reviewers (authors NA, TKL, KW, GL YA, and BAB) independently screened studies for eligibility in the title and abstract stage as well as the full-text review stage. We first examined studies by title and abstract. After removing studies that did not meet the inclusion criteria, we screened the full text of the remaining articles again for eligibility. Conflicts were resolved through discussion among the reviewers or by a third reviewer when necessary. With the eligible studies, we then assessed the risk of bias and extracted data. All references within the included studies were checked to identify additional relevant articles.

### Risk of bias assessment

The risk of bias was assessed in accordance with the Consensus Health Economic Criteria (CHEC) list guidelines [30]. The CHEC tool includes 19 criteria, based on study design, for items ranging from a clear description of competing alternatives to the appropriate measurement of costs and value of outcomes to inform how well an article has addressed the minimum quality elements of an economic evaluation. Studies that achieved equal to lower than 80% (< 16 out of 19) of the CHEC list were considered to be at high risk of bias and of low quality and therefore excluded from our review. We reasoned that implementing a cut-off score in the quality assessment process is especially critical for a systematic review that focuses on cost-effectiveness analyses—as studies without relevant information to provide context renders the results opaque and difficult to evaluate. Details of our risk bias assessment are presented in Table 1.

**Table 1:** CHEC list grading

Author (Year)	Chalder et al. 2012	Gulliford et al. 2014	Gusi et al. 2008	Kraepelien et al. 2018	Kuo et al. 2021	Murphy et al. 2012	Philipsson et al. 2013	Priebe et al. 2016	Sun et al. 2021	Taylor et al. 2020	Turner et al. 2017	Underwood et al. 2013	van Eeden et al. 2015
**Is the study population clearly described?**	Y	Y	Y	Y	Y	Y	Y	Y	Y	Y	Y	Y	Y
**Are competing alternatives clearly described?**	Y	Y	Y	Y	Y	Y	Y	Y	Y	Y	Y	Y	Y
**Is a well-defined research question posed in answerable form?**	Y	Y	Y	Y	Y	Y	Y	Y	Y	Y	Y	Y	Y
**Is the economic study design appropriate to the stated**	Y	Y	Y	Y	Y	Y	Y	Y	Y	Y	Y	Y	Y
**Is the chosen time horizon appropriate in order to include**	Y	Y	Y	Y	Y	Y	Y	Y	Y	Y	Y	N/A	Y
**Is the actual perspective chosen appropriate?**	Y	N/A	Y	Y	Y	Y	Y	Y	Y	Y	Y	Y	Y
**Are all important and relevant costs for each alternative**	Y	N	Y	Y	Y	Y	Y	Y	Y	Y	Y	Y	Y
**Are all costs measured appropriately in physical units?**	Y	N/A	Y	Y	Y	Y	Y	Y	Y	Y	Y	Y	Y
**Are costs valued appropriately?**	Y	N/A	Y	Y	Y	Y	Y	Y	Y	Y	Y	Y	Y
**Are all important and relevant outcomes for each alternative**	Y	Y	Y	Y	Y	Y	Y	Y	Y	Y	Y	Y	Y
**Are all outcomes measured appropriately?**	Y	Y	Y	Y	Y	Y	Y	Y	Y	Y	Y	Y	Y
**Are outcomes valued appropriately?**	Y	Y	Y	Y	Y	Y	Y	Y	Y	Y	Y	Y	Y
**Is an incremental analysis of costs and outcomes of alternatives**	Y	Y	Y	Y	N/A	Y	Y	Y	Y	Y	Y	Y	Y
**Are all future costs and outcomes discounted appropriately?**	Y	Y	Y	Y	N/A	N/A	Y	N/A	N/A	N/A	Y	N/A	Y
**Are all important variables**,** whose values are uncertain**,	Y	Y	Y	N	Y	Y	Y	Y	Y	Y	Y	N/A	Y
**Do the conclusions follow from the data reported?**	Y	Y	Y	Y	Y	Y	Y	Y	Y	Y	Y	Y	Y
**Does the study discuss the generalizability of the results to**	Y	Y	Y	Y	Y	Y	Y	Y	N	Y	Y	N	N
**Does the article indicate that there is no potential conflict of**	N	N	N	Y	Y	Y	Y	Y	Y	Y	Y	N	Y
**Are ethical and distributional issues discussed appropriately?**	N	N	Y	N	N	N	Y	N	N	Y	N	N	Y
**TOTAL SCORE (OUT OF 19)**	17	13	18	17	16	17	19	17	16	18	18	13	18
**% achieved**	89	68	95	89	84	89	100	89	84	95	95	68	95

### Data extraction and analysis

Key details were extracted from studies using a predetermined 32-item matrix, including basic information such as title and author names, in the Covidence software platform. A summary of key data extracted from each study included in our review is provided in Table 2. Study outcomes were summarized in a narrative synthesis.

**Table 2:** Details of included studies

Study	Study characteristics		Results
	Descriptive characteristics	Technical charcteristics	
Chalder 2012	Perspective: Healthcare Intervention/Comparator: Physical activity intervention, in addition to usual general practitioner care Simulated population: Aged 18–69 years with an International Statistical Classification of Diseases and Related Health Problems, 10th Edition (ICD-10) diagnosis of depression and scoring ≥ 14 on the Beck Depression Inventory (BDI). Country/Currency (adj. year): UK; GBP (2009)	Modeling approach: Time horizon: 1 year Discounting: 3.5 Threshold used: £20,000 and £30,000/QALY	Results: £19,394/QALY gained Author’s conclusions: This physical activity intervention is very unlikely to lead to any clinical benefit in terms of depressive symptoms or to be a cost-effective treatment for depression. % EE assessment satisfied: 89%
Gusi 2008	Perspective: Healthcare Intervention/Comparator: Two alternatives: best care in general practice and the addition of a walking programme were compared. Simulated population: women were aged 60 yearsand older, suffered from either moderate depression orwere overweight, and were capable of walking for morethan 25 min. Country/Currency (adj. year): Spain; Euro (2005)	Modeling approach: Time horizon: 6 months Discounting: None Threshold used: €34,729/QALY	Results: €311/QALY Author’s conclusions: The current study presented a pragmatic and cost-effective strategy to enhance the level of physical activity in over-weight or moderately depressed elderly women; the programme could be a cost-effective resource for helping patients increase physical activity, as recommended by general practitioners. % EE assessment satisfied: 95%
Kraepelien 2018	Perspective: Healthcare and societal perspectives Intervention/Comparator: Both internet-based cognitive–behavioural therapy (ICBT) and physical exercise as alternatives to treatment as usual (TAU) Simulated population: Aged 18–67 years and present depressive symptoms defined as scoring ≥ 10 on the Patient Health Questionnaire (PHQ-9) Country/Currency (adj. year): Sweden; USD (2012)	Modeling approach: Time horizon: 1 year Discounting: None Threshold used: €21 536 (£20 000)/QALY	Results: €14 571/QALY gained Author’s conclusions: From a primary care perspective, both ICBT and physical exercise for depression are likely to be cost-effective compared with TAU. % EE assessment satisfied: 89%
Kuo 2021	Perspective: Healthcare and societal perspectives Intervention/Comparator: Program ACTIVE (Adults Coming Together to Increase Vital Exercise) II community-based exercise (EXER), cognitive behavioral therapy (CBT), and EXER + CBT interventions. Simulated population: The mean age of the simulated population was 57.3 years, 24% were male, 70.7% were White, mean type 2 diabetes duration was 13.3 years, and mean BMI was 36.6 kg/m2. Country/Currency (adj. year): United States; USD (2014)	Modeling approach: Time horizon: 10 years Discounting: 3 Threshold used: Not reported	Results: Exercise alone- Dominates Exercise + CBT- 600/QALY gained Author’s conclusions: All three Program ACTIVE II interventions represented a good value for money compared with UC; the EXER + CBT intervention was highly cost-effective or cost saving compared with the CBT or EXER interventions. % EE assessment satisfied: 84%
Murphy 2012	Perspective: Public sector Intervention/Comparator: NERS vs. leaflet and normal care Simulated population: Participants were aged between 16 and 88 years (mean 52, SD14.7), predominately women (66%) and the vast majority classed themselves as white (96%). Country/Currency (adj. year): UK; GBP (NR)	Modeling approach: Time horizon: 1 year Discounting: None Threshold used: £20 000-£30 000/ QALY	Results: £12,111/QALY Author’s conclusions: NERS was effective in increasing physical activity among those referred for CHD risk only; among mental health referrals, NERS did not influence physical activity but was associated with reduced anxiety and depression, and the effects were dependent on adherence. % EE assessment satisfied: 89%
Philipsson 2013	Perspective: Societal Intervention/Comparator: The intervention comprised dance twice weekly during eight months in addition to usual school health services vs. usual school health services Simulated population: Adolescent girls 13–18 years old with internalizing problems Country/Currency (adj. year): Sweden; USD (2011)	Modeling approach: Time horizon: 1 year Discounting: 3 Threshold used: $75,000/QALY	Results: $3,830/QALY gained Author’s conclusions: Intervention with dance twice weekly in addition to usual school health services may be considered cost-effective compared with usual school health services alone, for adolescent girls with internalizing problems. % EE assessment satisfied: 100%
Priebe 2016	Perspective: Health and social care Intervention/Comparator: Psychotherapy group vs. Pilates group Simulated population: Age 18–65 years; diagnosis of schizophrenia with symptoms present at > 6 months; score of ≥ 18 on Positive and Negative Syndrome Scale (PANSS) negative symptoms subscale Country/Currency (adj. year): UK; GBP (2014)	Modeling approach: Time horizon: Not reported Discounting: Not reported Threshold used: Not reported	Results: £119/QALY gained Author’s conclusions: In comparison with an active control, group body psychotherapy does not have a clinically relevant beneficial effect in the treatment of patients with negative symptoms of schizophrenia. % EE assessment satisfied: 89%
Sun 2021	Perspective: Healthcare Intervention/Comparator: Behavioral activation with mindfulness (BAM) vs. care as usual (CAU). Simulated population: Participants were included if they were aged 18 years or above, could speak Cantonese, had subthreshold depression defined as a Patient Health Questionnaire-9 score over 4. Participants were excluded if they had dysthymia, major depression within the past 6 months, lifetime history of other psychiatric disorders, presence of serious suicidal risk, medical illness with a prognosis of less than 6 months to live, or were currently receiving treatments for depression. Country/Currency (adj. year): Hong Kong - China; HKD (2015)	Modeling approach: Time horizon: 1 year Discounting: None Threshold used: $50,000/QALY	Results: $5,979/QALY gained Author’s conclusions: Group-based BAM is considered as a cost-effective alternative treatment for treating subthreshold depression by preventing major depressive disorder. % EE assessment satisfied: 84%
Taylor 2020	Perspective: Health and social care Intervention/Comparator: e-coachER vs. control Simulated population: Aged 16–74 years, with a body mass index of 30–40 kg/m2, with hypertension, prediabetes, type 2 diabetes, lower limb osteoarthritis or a current/recent history of treatment for depression, who were also inactive, contactable via e-mail and internet users. Country/Currency (adj. year): UK; GBP (2018)	Modeling approach: Time horizon: 1 year Discounting: None Threshold used: £30,000/QALY	Results: £9000 - £15,885/ QALY gained Author’s conclusions: Adding e-coachER to usual exercise referral schemes had only a weak indicative effect on long-term rigorously defined, objectively assessed moderate and vigorous physical activity. % EE assessment satisfied: 95%
Turner 2017	Perspective: Health and social care Intervention/Comparator: The intervention comprised 12 separate sessions of circuit training over a 6-week period. Participants also received treatment as usual. The comparator group received treatment as usual. Simulated population: Aged 14–17 years attending Tier 2 and Tier 3 CAMHS (Child and Adolescent Mental Health Services) outpatient services presenting with depression. Country/Currency (adj. year): UK; GBP (2013)	Modeling approach: Time horizon: <1 year Discounting: None Threshold used: Not reported	Results: £152 822/ QALY Author’s conclusions: There is evidence that exercise can be an effective intervention for adolescents with depression and the current study shows that preferred intensity exercise could also represent a cost-effective intervention in terms of the CDI-2. % EE assessment satisfied: 95%
vanEeden 2015	Perspective: Societal Intervention/Comparator: Augmented CBT vs. CogniPlus Simulated population: Stroke patients (aged 18 + years) with signs of depression Country/Currency (adj. year): The Netherlands; Euro (2012)	Modeling approach: Time horizon: 1 year Discounting: None Threshold used: 40,000	Results: Dominated Author’s conclusions: Taking into account the limitations of the current study, we conclude that the preliminary results of the cost-effectiveness of the stroke-specific augmented CBT intervention under investigation were not convincing. % EE assessment satisfied: 95%

## Findings

**Fig. 1:**
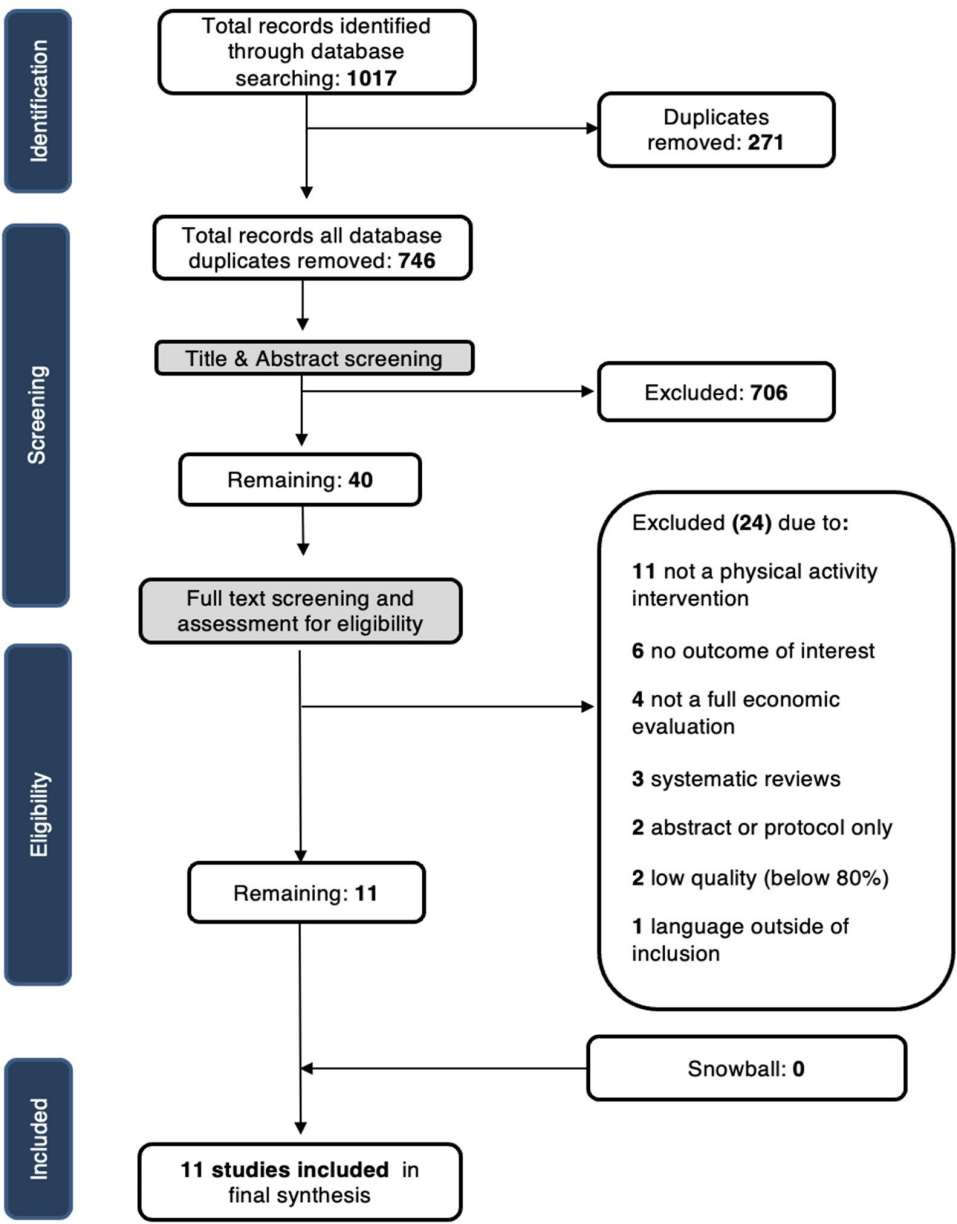
PRISMA diagram of screening results

The initial database searches identified 1,017 references. Of the initial search references, 271 duplicates were removed, leaving 746 references for review during the initial round of screening of titles and abstracts. During the title and abstract review, 706 studies were ineligible for inclusion, and 40 studies underwent full-text screening. Inter-rater reliability (IRR) was high during title and abstract screening (0.77–1.00). During full-text screening, 11 studies did not assess a physical activity intervention, and six studies did not report on outcomes of interest; seven studies were not full economic evaluations, of which three were systematic reviews. The three systematic review studies were excluded, and the reviewers hand-searched the reference lists of these articles for potential studies. Substantial agreement was maintained during full-text review with an IRR of (0.66–1.00). Two studies were available as abstracts only, and one study was not published in English. Two studies that met the inclusion criteria were excluded from the final analysis due to low quality as determined by the CHEC risk of bias assessment step. Underwood et al. 2013 did not include appropriate future discounting or sensitivity analyses, and Gulliford 2014 lacked key information regarding relevant costs for each alternative identified [31, 32]. Following the screening steps, 11 studies were included in our final review (Fig. 1).

### Descriptive characteristics

We extracted eight descriptive characteristics of focus from the included economic evaluation studies. Summaries are presented below.

#### Perspective chosen

A majority of included studies conducted the CEA or CUA from only a public sector or healthcare perspective (7 studies; 64%). Two studies used only a societal perspective (18%). Another two studies used both a healthcare perspective and a societal perspective (18%).

#### Intervention and comparator

Most models assessed interventions based on group exercise sessions (6 studies; 55%). Group sessions included activities such as a supervised walking-based exercise program (three 50-minute sessions per week), group exercise sessions (three times per week), twice weekly 75-minute dance classes, 60-minute circuit training sessions twice a week, or body psychotherapy. One-on-one exercise support was assessed in three models (27%). One-on-one interventions included a referral to the National Exercise Referral Scheme (NERS), recommending 30 min of moderate physical activity at least 5 days per week, access to web-based coaching support packages, or face-to-face and telephone contact with trained physical activity facilitators. A majority of the models compared the interventions in addition to usual services against the standard of care or no new intervention (8 studies; 73%). However, three of the models (27%) evaluated the cost-effectiveness of the intervention of interest by comparing it against another active comparator option that would change the standard of care [33–35]. 

#### Setting and study population

Four studies assessed interventions in the general practice setting [36–39], while three studies assessed community-based interventions, such as adult community-based group exercise [33, 34, 40]. Two studies identified the setting of the assessment as an urban setting [41, 42], and one study assessed an intervention in a rehabilitation center [35]. The final study did not specify a setting [43]. 

Most models evaluated the cost-effectiveness of the intervention when administered to adult populations presenting with a disorder such as depression or schizophrenia (8 studies; 73%). Two studies modeled interventions amongst adolescent populations (18%) [40, 41], and one study focused on women 60 years and older [37]. 

#### Targeted mental health conditions

The interventions included in our review targeted a number of mental disorders. The most common condition was depression (10 studies; 91%). Four of these studies targeted depression simultaneously with other conditions such as obesity, diabetes, and heart conditions. A single study focused on patients with schizophrenia [34]. 

#### Country and currency

Most of the models identified in our review were conducted in the United Kingdom (UK) (5 articles; 45%), with costs expressed in Great British Pounds (GBP). Studies from Sweden (2 studies; 18%) converted local currency (Swedish Krone) to the United States Dollar (USD). Two studies were based in European countries, including The Netherlands (*n* = 1) and Spain (*n* = 1), and demonstrated costs as Euros. Studies from Hong Kong (*n* = 1) and the USA (*n* = 1) conveyed costs in local currency.

#### Cost estimations and effectiveness outcome measure

A majority of the studies (10 studies; 91%) only considered the direct costs of the intervention, such as session materials, and costs for trainers, therapists, or nurses. A single study considered both direct and indirect costs such as healthcare procedures and medications, and loss of productivity at work [38]. All studies identified in our review used quality-adjusted life years (QALYs) as the effectiveness outcome measure (11 articles; 100%).

### Technical characteristics

Five technical characteristics extracted from included studies are outlined in detail below.

#### Study design

Nearly all studies included in our review were CEAs conducted in parallel with randomized controlled trials (RCT) (10 studies; 91%). Although conducted alongside RCTs, these CEAs included a modeling component (instead of only a incremental cost-effectiveness ratio without modeling calculation, which used true observed results). Modeling indicates the creation of a simplified representation of the real world to aid decision-making. A single study used a microsimulation study design without being part of an RCT.

#### Time horizon

Time horizons to account for cost and outcomes used by the models ranged between 6 months to 10 years. A one-year time horizon was the most commonly used (*n* = 6; 55%) among this set of studies. One study did not report any detail related to the use of a time horizon.

#### Discounting

Discounting captures the loss in economic value due to a delay in receiving realized benefits. Selected discount rates ranged from 3.00 to 3.50%. Seven studies (64%) used time horizons of 1 year or less and, therefore, did not apply discounting. One study did not report any detail related to the use of a discount rate.

#### Sensitivity analysis

Sensitivity analyses test the parameters of model variables either by means of deterministic (i.e., test parameters by changing one value at a time while all other parameters remain constant) or probabilistic (i.e., test by assigning probability distributions to parameters and simulating many inputs at once) analyses. Of the included studies, only four performed deterministic sensitivity analysis. Two studies (18%) conducted both deterministic and probabilistic sensitivity analyses. Two studies (18%) did not perform sensitivity analyses, and it was unclear if sensitivity analyses were conducted for an additional two studies (18%).

#### Cost-effectiveness threshold

Thresholds determine the level at which interventions would be considered as cost-effective. The standard National Institute for Health and Care Excellence (NICE) UK threshold of £20,000–30,000 per QALY gained was the most common (4 studies; 36%). In the US setting, a threshold of $50,000 per QALY gained was utilized. Other thresholds included €40,000 per QALY gained and $75,000 per QALY gained. Three studies either did not apply a threshold or did not report one in their article.

### Cost-effectiveness of interventions

Cost-effectiveness of interventions can be represented by the incremental cost-effectiveness ratio (ICER), which simply summarized, is the difference in costs divided by the difference in outcome (∆Cost/∆Effect). The ICER captured in this review ranged from £119 to £152 822 per QALY gained (Table 2). In one case, a cognitive behavioral intervention augmented with occupational and movement therapy dominated a computerized cognitive training program [35]. 

While most studies generated an ICER (with only two studies indicating that their intervention was dominated), the authors from over half of all studies concluded that their respective examined interventions were found not to be cost-effective substantively (6 articles; 55%). These conclusions are primarily driven by uncertainties about the clinical benefit of the interventions – as noted by the authors. Due to small and uncertain incremental benefits on QALYs gained, the cost per QALY calculations indicated a low probability that the interventions are cost-effective relative to the standard of care. However, five (45%) articles identified interventions that demonstrated cost-effectiveness, including studies on group exercise-based interventions such as walking, dance, or community-based exercise, mindfulness sessions, and referral programs for exercise support. We compared results that analyzed similar interventions and alternatives to summarize the evidence related to intervention cost-effectiveness.

#### Group exercise sessions

The most common intervention type was group exercise sessions (6 studies; 55%) [33, 34, 37, 38, 40, 41]. All programs met consistently on a weekly basis. The evidence related to these group sessions was mixed. Although dance sessions, walking programs, and community-based group exercises were found to be cost-effective, neither circuit training for adolescents with depression nor Pilates for patients with schizophrenia were found to be cost-effective.

#### One-on-one exercise support

Individualized support and guidance to help patients achieve physical activity goals and targets was the second most commonly reported intervention (3 studies; 27%). Ongoing guidance and support delivered by exercise professionals were also provided through one-on-one consultations, which were occasionally delivered via telephone or web-based. A majority of these one-on-one support interventions (2 studies; 67%) were found to be cost-effective [42, 43]. However, in one instance, the intervention was expected not to result in clinical benefits for depressive symptoms; therefore, this intervention was likely not a cost-effective treatment [36]. 

Other interventions included individual movement therapy for stroke patients with depression [35] and weekly mindfulness sessions delivered by trained allied healthcare workers [39]. Although the movement therapy sessions were not found to be cost-effective, group-based mindfulness was considered a cost-effective alternative treatment to prevent major depressive disorder.

## Discussion

This review described and summarized published CEAs on physical activity-oriented interventions for improving mental health across a range of conditions. These interventions included elements such as group exercise sessions, one-on-one exercise planning and support, movement therapy, and mindfulness exercise to address mental health conditions, including depression, anxiety, and stress. Eleven high-quality studies that analyze the cost-effectiveness of such interventions were identified.

Our review finds that the current evidence is insufficient to come to strong conclusions about whether physical activity-oriented interventions for mental health are cost-effective when compared with the standard of care of other treatment types. However, physical activity-oriented interventions that are relatively low cost, such as telephone or web support for exercise goals and motivation, yielded results that were cost-effective. In addition, the most commonly used outcome measure in health—QALYs—may be ill-fitting when applied to mental health conditions [44]. This pattern can be particularly problematic—as instruments to determine QALYs are dominated by physical health indicators [45]. Importantly, based on the evidence presented, we were unable to draw any conclusions regarding productivity-related costs, such as absenteeism and presenteeism.

Heterogeneity of the available evidence limited the capacity to determine specific successful interventions; however, we identified several program elements that may contribute to variability in cost-effectiveness. One variable is the duration and frequency of the activities of the intervention groups between the studies that found cost-effectiveness versus those that did not. Four studies that observed cost-effectiveness had consistent intervention duration between 12 weeks and up to 8 months with 2 to 5 interventions per week [37, 38, 41, 43]. In contrast, four studies that did not observe cost-effectiveness had intervention duration between 1 and 10 weeks [33, 42] or between 10 and 13 sporadic intervention sessions spread out over the course of 4 to 8 months [35, 36]. Two studies also noted cost-effectiveness despite finding no difference in improving QALY between intervention and control trials, with intervention durations of 6 and 8 weeks [39, 40]. The difference in duration, frequency, and regularity between the studies that noted cost-effectiveness and those that did not was striking and help explain the discrepancy noted. Future studies may wish to examine characteristics of intervention in the context of duration and frequency of intervention, as well as intensity of physical activity. Focusing on regular and sustained physical activity over time may increase intervention effectiveness and improvement in QALY.

Furthermore, adding to the uncertainty regarding the cost-effectiveness of interventions evaluated in this study, all interventions were delivered for a limited period, and there remains a high uncertainty regarding the real-world, long-term benefits of the programs once sessions are no longer delivered. Additional follow-up of interventions is necessary to provide robust inputs on the long-term effect of models. Models in our review often only accounted for costs and outcomes over a one-year period; therefore, it was difficult to capture and determine if there were any considerable benefits of these interventions beyond this period. Future CEAs may also want to consider the long-term health gains from physical activity-oriented interventions.

Evaluations identified in this study rarely considered other important costs, such as productivity cost and cascading healthcare services, which may be influenced by the intervention. As such, it is critical to note the limitation in the scope of summarized results.

Lastly, physical activity-oriented interventions for improving mental health conditions are reasoned to incur opportunity costs, which are defined as the benefit forgone when seeking care. These activities require time participants could spend engaging in other activities: however, these costs are difficult to quantify and rarely included in economic evaluations of physical activity interventions [46, 47]. Such opportunity costs should be documented in future CEAs and compared with the opportunity costs of pharmacological interventions (e.g., time lost due to side effects). Numerous reviews have shown that use of very modest incentives can sustain increases in physical activity over time, even once the incentives have ceased [48, 49]. Therefore, the relevance of time in such interventions makes incentivizing quality physical activity and exercise significantly more important. That is, the cost could be significantly reduced, and benefits significantly increased, if an intervention can incentivize higher benefits per minute spent engaging in physical activities.

In summary, our review identified two main mechanisms by which physical activity-oriented interventions that aim to improve mental health may yield improved cost-effectiveness. The first mechanism is to ensure that the economic costs of these interventions remain low. This element requires considering a lower cost mode of delivery (e.g., platform used to deliver physical activity instructions) and taking into consideration the opportunity cost of participants (e.g., time required to engage in physical activities). A second is to adopt a behavioral economic component to incentivize sustainable, higher quality, and higher intensity (when appropriate and feasible) exercise. Behavioral economic interventions are designed to address existing cognitive biases in human behavior, such as using financial incentives, simplifying appointment making, and using nudges. Such incentive may be a low-cost mechanism to yield increased effectiveness, making an intervention more cost-effective.

### Limitations

Published cost-effectiveness studies have the tendency to present cost-effective interventions (instead of null results and non-cost-effectiveness interventions); we recognize that such a pattern may lead to bias in systematic reviews. Nevertheless, in our study, we found that over half of the studies did not find the intervention evaluated to be cost-effective. Included studies have a strong geographical bias towards high-income countries; this pattern is likely due to the resources available for research studies, data collection, as well as human capital available to conduct cost-effectiveness analyses that are often time intensive. Recognizing these limitations, we were careful to evaluate and document in detail the quality of studies that provided robust evidence to support cost-effectiveness of physical activity-oriented interventions. In this process, we did not evaluate the instruments that were used to generate QALY and note this as a limitation of the study. We hope these details may enable researchers and policymakers to determine if the findings can be generalized to other settings. In addition, most studies adopted a narrow perspective in their analysis, which limits the generalizability.

## Conclusions

Our review identified 11 studies on the cost-effectiveness of physical activity-oriented interventions for improving mental health. Although approximately half of the identified studies analyzed found the intervention of interest not cost-effective compared to the standard of care or an active comparator. The lack of efficacy may be partially due to the quality of physical activity and exercise that each intervention was able to derive from participants. Given the study findings on the efficacy of these interventions, it is logical that only especially low-cost physical activity-oriented interventions were able to yield a favorable incremental cost-effectiveness ratio. Moving forward, we encourage physical activity-oriented interventions that aim to improve mental health to adopt low-cost implementation strategies and include behavioral economics components to incentivize better quality and sustained physical activity and exercise.

## Electronic supplementary material

Below is the link to the electronic supplementary material.

Supplementary Material 1

## Data Availability

No datasets were generated or analysed during the current study.

## Declarations

### Disclaimer

The findings, interpretations, and conclusions expressed in this work are those of the authors, and do not necessarily reflect the views of King Faisal Specialist Hospital and Research Center or the World Bank, their Boards of Directors, or the governments they represent.

## Abbreviations

CHEC: Consensus on Health Economic Criteria
CBT: Cognitive behavioral therapy
GBP: Great British Pounds
ICER: Incremental cost effectiveness ratio
NICE: National Institute for Health and Care Excellence
PRISMA: Preferred Reporting Items for Systematic Reviews and Meta-Analyses
QALY: Quality-adjusted life year
UK: United Kingdom
US: United States
USD: United States Dollar
