# Supplementary material for: Cost-effectiveness of physical activity-oriented interventions for improving mental health: a systematic review
Source: BMC Public Health. 2025 May 13;25:1766. doi: 10.1186/s12889-025-22207-3 (PMC12070520; doi:10.1186/s12889-025-22207-3)
Supplement: Supplementary file 1 — Supplementary Material 1 [file 12889_2025_22207_MOESM1_ESM.docx]

**Supplementary Table S1. Search Strategy**

All searches conducted November 29, 2022

| **PubMed – 687 studies** | |
| --- | --- |
| **1** | (((((stress disorders, traumatic[MeSH Terms])OR (bipolar depression[MeSH Terms])) OR (depression[MeSH Terms])) OR (anxiety disorders[MeSH Terms])) OR (dementia[MeSH Terms])) OR (schizophrenia[MeSH Terms]) |
| **2** | "depression"[Title/Abstract] OR "anxiety"[Title/Abstract] OR "anxiety disorder"[Title/Abstract] OR "dementia"[Title/Abstract] OR "PTSD"[Title/Abstract] OR "post-traumatic stress disorder"[Title/Abstract] OR "posttraumatic stress disorder"[Title/Abstract] OR "schizophrenia"[Title/Abstract] OR "bi-polar disorder"[Title/Abstract] |
| **3** | mental health disorders[MeSH Terms] |
| **4** | #1 OR #2 OR #3 |
| **5** | "Cost-Benefit Analysis"[MeSH Terms] OR "cost-effectiveness analysis"[Title/Abstract] OR "CEA"[Title/Abstract] OR "cost-utility analysis"[Title/Abstract] OR "CUA" [Title/Abstract] OR "cost-benefit analysis" [Title/Abstract] OR "CBA" [Title/Abstract] OR "economic evaluation*"[Title/Abstract] |
| **6** | (((((activities, physical[MeSH Terms]) OR (physical activity[MeSH Terms])) OR (aerobic exercise[MeSH Terms])) OR (sports[MeSH Terms])) OR (bicycling[MeSH Terms])) |
| **7** | "exercise*"[Title/Abstract] OR "physical activit*"[Title/Abstract] OR "physical fitness"[Title/Abstract] OR "walk*"[Title/Abstract] OR "runn*"[Title/Abstract] OR "swim*"[Title/Abstract] OR "jog" [Title/Abstract] OR "jogging" [Title/Abstract] OR "train*"[Title/Abstract] OR "aerobic*"[Title/Abstract] OR "anerobic*"[Title/Abstract] OR "cycl*"[Title/Abstract] OR "danc*"[Title/Abstract] OR "yoga"[Title/Abstract] |
| **8** | #6 OR #7 |
| **9** | "association"[MeSH Terms] OR "risk factors" [MeSH Terms] |
| **10** | "protect*"[Title/Abstract] OR "prevent*"[Title/Abstract] OR "relation*"[Title/Abstract] OR "causa*"[Title/Abstract] OR "onset"[Title/Abstract] |
| **11** | #9 OR #10 |
| **12** | letter[pt] OR editorial[pt] OR conference review [pt]OR published erratum [pt] OR case reports [pt] OR interview[pt] |
| **13** | #4 AND #5 AND #8 AND #11 |
| **14** | #13 NOT #9 |
|  |  |
| **Science Direct – 22 studies** | |
|  | (mental health disorders OR dementia) AND (cost-effectiveness analysis OR economic evaluation) AND (exercise OR physical activity) |
|  |  |
| **PsychArticles – 2 articles** | |
| **1** | MA stress disorders, traumatic OR bipolar depression OR depression OR anxiety disorders OR dementia OR schizophrenia |
| **2** | AB depression OR anxiety OR anxiety disorder OR dementia OR PTSD OR post-traumatic stress disorder OR posttraumatic stress disorder OR schizophrenia OR bi-polar disorder |
| **3** | S1 OR S2 |
| **4** | MA Cost-Benefit Analysis |
| **5** | AB cost-effectiveness analysis OR cost-utility analysis OR cost-benefit analysis OR economic evaluation* |
| **6** | S4 OR S5 |
| **7** | MA activities, physical OR physical activity OR aerobic exercise OR sports OR bicycling |
| **8** | AB exercis* OR physical activit* OR walk* OR runn* OR swim* OR jog OR jogging OR train* OR aerobic* OR anerobic* OR cycl* OR danc* OR yoga |
| **9** | S7 OR S8 |
| **10** | S3 AND S6 AND S9 |
|  |  |
| **PsychINFO – 142 studies** | |
| **1** | MA stress disorders, traumatic OR bipolar depression OR depression OR anxiety disorders OR dementia OR schizophrenia |
| **2** | AB "depression" OR "anxiety" OR "anxiety disorder" OR "dementia" OR "PTSD" OR "post-traumatic stress disorder" OR "posttraumatic stress disorder" OR "schizophrenia"OR "bi-polar disorder" |
| **3** | S1 OR S2 |
| **4** | MA Cost-Benefit Analysis |
| **5** | AB "cost-effectiveness analysis" OR "cost-utility analysis" OR "cost-benefit analysis" OR "economic evaluation*" |
| **6** | S4 OR S5 |
| **7** | MA activities, physical OR physical activity OR aerobic exercise OR sports OR bicycling |
| **8** | AB "exercis*" OR "physical activit*"OR "walk*" OR "runn*" OR "swim*" OR "jog" OR "jogging" OR "train*" OR "aerobic*" OR "anerobic*" OR "cycl*" OR "danc*" OR "yoga" |
| **9** | S7 OR S8 |
| **10** | S3 AND S6 AND S9 |
| **11** | MA mice OR animal sex differences OR animals |
| **12** | 10 NOT 11 |
|  |  |
| **Web of Science – 164 studies** | |
| **1** | AB= ("traumatic stress disorders*" OR "bipolar depression" OR "depression" OR "anxiety disorder*" OR "dementia" OR "schizophrenia" OR "anxiety" OR "PTSD" OR "post-traumatic stress disorder" OR "posttraumatic stress disorder" OR "bi-polar disorder" OR "mental health disorder*") |
| **2** | AB = ("cost-effectiveness analysis" OR "cost effectiveness analysis" OR "cost-utility analysis" OR "cost-benefit analysis" OR "economic evaluation*") |
| **3** | AB = ("physical activity" OR "exercise" OR "physical fitness" OR "aerobic exercise" OR "sport*" OR "bicycling" OR "exercis*" OR "physical activit*"OR "walk*" OR "runn*" OR "swim*" OR "jog" OR "jogging" OR "train*" OR "aerobic*" OR "anerobic*" OR "cycl*" OR "danc*" OR "yoga") |
| **4** | AB = ("association" OR "risk factors" OR "protect*"OR "prevent*" OR "relation*" OR "causa*" OR "onset") |
| **5** | #1 AND #2 AND #3 |
| **6** | #5 NOT DT=(Note OR Letter OR Meeting Abstract OR News Item OR Editorial Material) |

Appendix 1. List of data extraction items

- Study details
  - Title
  - Lead Author
  - Year of publishing
  - Country of origin
- Methods
  - Aim of study
  - Study design
  - Setting
  - Intervention and comparators
  - Activity intensity of the interventions
  - Participants
    - Population description
    - Total number of participants
    - Disease targeted
  - Technical characteristics
    - Perspective
    - Currency (year of cost data)
    - Discount rate
    - Time horizon
    - Source of cost data
    - Source of effectiveness data
    - Health outcomes measured
    - Types of costs collected
    - Threshold used
    - Sensitivity analyses conducted
  - Key findings
    - Results
    - Incremental costs
    - Incremental effectiveness
    - ICER
    - Authors relevant findings
    - Other info
    - Notes
